# Recent advances in nanorobotic manipulation inside scanning electron microscopes

**DOI:** 10.1038/micronano.2016.24

**Published:** 2016-06-20

**Authors:** Chaoyang Shi, Devin K Luu, Qinmin Yang, Jun Liu, Jun Chen, Changhai Ru, Shaorong Xie, Jun Luo, Ji Ge, Yu Sun

**Affiliations:** 1 Department of Mechanical and Industrial Engineering, University of Toronto, Toronto, ON, Canada M5S 3G8; 2 Department of Control Science and Engineering, Zhejiang University, Hangzhou 310058, China; 3 Robotics and Microsystems Center, Soochow University, Suzhou 215021, China; 4 School of Mechatronic Engineering and Automation, Shanghai University, Shanghai 200072, China

**Keywords:** automated nanomanipulation, scanning electron microscope, SEM-based nanomanipulation

## Abstract

A scanning electron microscope (SEM) provides real-time imaging with nanometer resolution and a large scanning area, which enables the development and integration of robotic nanomanipulation systems inside a vacuum chamber to realize simultaneous imaging and direct interactions with nanoscaled samples. Emerging techniques for nanorobotic manipulation during SEM imaging enable the characterization of nanomaterials and nanostructures and the prototyping/assembly of nanodevices. This paper presents a comprehensive survey of recent advances in nanorobotic manipulation, including the development of nanomanipulation platforms, tools, changeable toolboxes, sensing units, control strategies, electron beam-induced deposition approaches, automation techniques, and nanomanipulation-enabled applications and discoveries. The limitations of the existing technologies and prospects for new technologies are also discussed.

## Introduction

A variety of nanomaterials, such as nanotubes, nanowires, plasmonic and semiconductive nanomaterials, and two-dimensional materials, such as graphene and transition metal dichalcogenides, require their properties to be characterized to understand their behaviors and explore their applications in nanoelectronics, nanophotonics, biology, and medicine^1–5^. The fabrication and development of nanoscaled devices and nanoelectromechanical systems (NEMS) that use these nanomaterials also require precise techniques for positioning, sensing, and assembly with nanometer resolutions^3,6,7^.

Techniques for constructing nanoscaled devices can be categorized into top–down, bottom–up, and nanomanipulation-enabled techniques^3,7^. The top–down approaches typically employ techniques such as X-ray electron beam lithography and nanoimprint lithography^8–10^. Bottom–up techniques, such as self-assembly, chemical synthesis or super-molecule techniques^11,12^, are driven by the tendency of physical systems to minimize their potential energy. These techniques enable the construction of structures and devices via the direct assembly of atoms and molecules; however, modifying specific locations to adjust the device properties can be challenging. Nanorobotic manipulation is a complementary technique that enables flexible maneuvering and precise positioning of nanostructures for nano device assembly; it is particularly suitable for device prototyping and property tuning^3,13^. Papers that primarily focused on nanomanipulation applications and nanomanipulation inside an SEM were published in 2008, 2005, and previous years^8,13^. Conversely, this paper discusses technological advances and recent discoveries enabled by SEM-based nanomanipulation in addition to state-of-the-art applications.

The first nanorobotic manipulation experiment was performed by Eigler and Schweizer in 1990 (Ref. 14) with a scanning tunneling microscope (STM) to form the IBM logo by separately positioning 35 individual xenon atoms on a nickel substrate at low temperatures, which demonstrates atomic-level manipulation and fabrication. However, an STM requires the use of conductive probes and samples owing to its working principle^7^. To manipulate different types of materials, an atomic force microscope (AFM), which is a type of scanning probe microscope (SPM), is capable of subnanometer imaging and manipulation^15–17^. AFMs permit the manipulation of nanoscaled materials in ambient, aqueous, and vacuum environments, which render the technique capable of handling conductive, nonconductive, and biological materials^2,18^. However, performing simultaneous manipulation and imaging using AFMs remains challenging^13,17,19^ because the acquisition of one frame of an image by raster scanning requires at least several minutes. In addition, the look-then-move control scheme and small scanning area and workspace limit its manipulation throughput^7,17^.

A transmission electron microscope (TEM), which emits high-energy electrons that pass through a sample, is capable of subnanometer imaging. With the development of aberration correctors, state-of-the-art TEMs can achieve an imaging resolution of tens of picometers^20,21^ and perform atomic-level measurements for structural dynamics. However, the working principle of TEMs demands special sample preparation, and the small specimen chamber and workspace of a TEM limit the capabilities of complex nanomanipulation^13^. Conversely, the SEM detects secondary electrons that are emitted by the sample surface when struck by an electron beam, which provides real-time nanometer-level imaging^7^. Compared with TEMs, SEMs have substantially larger specimen chambers and scanning areas, which enable the integration of complex nanomanipulation systems. These advantages enable simultaneous imaging and precise nanomanipulation^3^.

A number of nanorobotic manipulation systems have been constructed for operation inside an SEM since the 1990s^8,13,22^. Hatamura and Tomomasa pioneered the development of a nanomanipulation system inside an SEM^23–25^. The system consisted of two manipulators, a controllable base, a customized force sensor, joy sticks, and an optical microscope, which enabled haptic and position control. Automated pick-and-place of 30 μm spheres inside an SEM was demonstrated^24^. The system was only capable of manipulating relatively large micrometer-sized objects and was not capable of performing complex assembly tasks owing to the mechanical wobbling of the manipulators and relatively large end-effectors.

Recently, nanorobotic manipulation platforms with multiple degrees of freedom (DOFs) and piezoelectric actuators have been constructed inside SEMs for manipulating nanoscaled objects with nanometer resolution, which enables the realization of complicated tasks that range from mechanical tensile testing and electrical probing of nanomaterials^26–30^, electronic and photonic device prototyping^1,22,31^, NEMS assembly^31–34^, biological cell characterization and manipulation^3,35,36^ and subcellular organelle extraction^35,37^. State-of-the-art SEM-based nanomanipulation systems are also integrated with AFMs and focused ion beam (FIB) systems, as well as various tools^38^ and exchangeable toolboxes^39^. With these advances, powerful nano-laboratories have been established that are capable of simultaneous imaging, fabrication, and nanomanipulation^40–43^ with high efficiency and reproducibility via the use of emerging automation techniques^30,44–46^.

## Nanomanipulation systems inside SEMs

Nanomanipulation systems inside SEMs primarily consist of an actuation unit, a sensing unit, control strategies, and nanotools. Techniques such as electron-beam-induced deposition (EBID) are often employed for nanoscaled welding and assembly. This section summarizes recent advances in these technologies.

### Actuation

Actuators that are employed for nanomanipulation inside an SEM must be compact, vacuum-compatible, and capable of generating accurate motions without interfering with electron microscopy imaging. Thermal actuators cannot effectively dissipate heat in a vacuum environment, whereas electric motors and voice coil actuators generate magnetic fields that can interfere with electron optics. Piezoelectric actuators that are governed by the inverse piezoelectric effect overcome these challenges^47,48^ and are capable of generating large forces in the kilo-Newton range with subnanometer positioning resolution and a high bandwidth^49^. A number of piezoelectric actuator-based nanomanipulators with multiple DOFs have been developed for precise positioning and manipulation of micro- and nanoscaled objects by companies (for example, Zyvex, Kleindiek, SmarAct, Toronto Nanoinstrumentation, and Attocube) and academia^33,41,42,50–54^.

**Figure 1:**
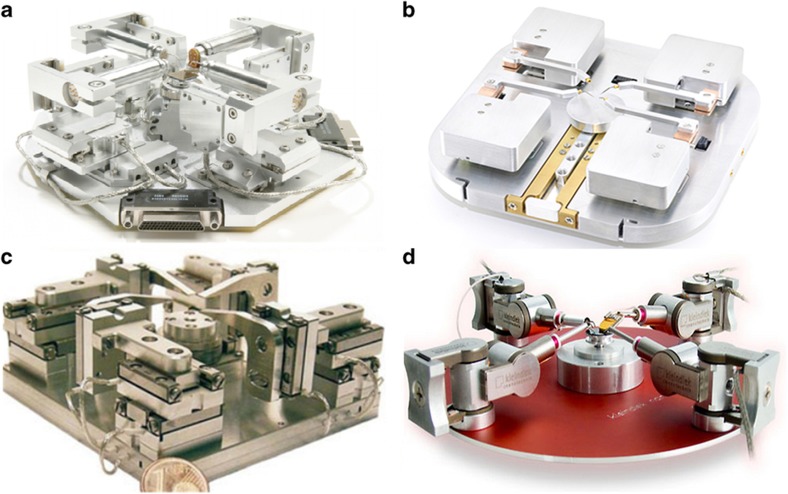
Examples of commercial nanomanipulation systems. (**a**) Zyvex. (**b**) Lifeforce. (**c**) SmarAct system. (**d**) Kleindiek.

The most common configuration of actuators for nanomanipulation is a coarse and fine positioner combination or a single actuator for both coarse and fine positioning. For instance, Zyvex and Lifeforce systems consist of four quadrants of three-DOF nanomanipulators, each of which uses two separate three-axis piezoelectric actuator units for coarse positioning and fine positioning, respectively, as shown in Figures 1a and b. The coarse positioners move according to the slip-stick actuation principle^49,55^ to generate stepwise motions at a high speed and with total motion ranges on the order of centimeters but with a low resolution and accuracy owing to unrepeatable steps. The separate fine unit uses piezo stacks, piezo tubes or flexure-guided mechanisms^47,55^ to precisely position the end-effector and enables a large fine motion range on the order of tens of micrometers (refer to Table 1). Kleindiek and SmarAct systems are composed of four quadrants of three-DOF nanomanipulators, each of which only uses one three-axis piezoelectric actuator unit. The single piezoelectric element either operates as a coarse actuator in the slip-stick mode or operates as a fine actuator solely in stick mode. This configuration produces a compact design but can induce undesired vibration during dynamic positioning, and the fine motion range is typically limited to <2 μm.

In addition to these stationary platforms, mobile nanomanipulators have also been developed^56–59^. Their actuation is typically based on the slip-stick principle^60^ or the inchworm mobile mechanism^59^ using piezoceramic actuators. These mobile platforms perform manipulation with nanometer accuracy. They collaborate among multiple robots and are capable of moving longer distances. However, the precise tracking of the position and orientation of the mobile robots is challenging. Conversely, stationary nanomanipulation platforms can generate more precise and accurate motion, are easier to control without localization and coordination difficulties, and facilitate automated nanomanipulation.

### Sensing

An important consideration for sensing in the vacuum environment is that heat generated from powering sensors cannot be effectively dissipated, which can cause instability owing to thermal drift. This section discusses advances in position, depth, and force sensing that are employed in SEM-based nanomanipulation.

#### Position sensing

Piezoelectric actuators inherently exhibit nonlinear characteristics of hysteresis and creep^49^, which degrade positioning precision and can cause system instability^61^. Three types of position sensors exist for integration with piezoelectric actuators, including capacitive, optical encoder-based and strain gauge-based sensors. Although piezoelectric actuators inherently possess sensing capabilities for estimating force and/or displacement^62^, piezoelectric self-sensing is difficult to implement in piezoelectric stacks, which is typically employed for the construction of nanopositioners.

Capacitive sensors provide a noncontact, low-power and direct position measurement approach for obtaining accurate nanometer and subnanometer measurements with a bandwidth up to 10 kHz and minimum heating^63^. However, capacitive sensors are costly and difficult for accurate assembly^64^ and are limited to short-range measurements within a couple of millimeters^65^. Optical encoder-based sensors generate incremental measurement readings that are capable of providing accurate position feedback for large-motion ranges (for example, tens of millimeters) at nanometer resolutions^41^. However, optical elements generate heat, which can cause thermal drift in surrounding components inside the vacuum chamber of an SEM^64^. Similarly, strain gauge-based sensors also suffer from thermal drift problems. When strain gauge-based sensors are utilized for position sensing inside an SEM, measuring the charging and discharging time of a capacitor that is connected to a strain gauge via time-to-digital conversion has been proven to generate 50 times less heat than optical encoders while achieving nanometer resolutions^64^.

#### Depth sensing

Height control in nanomanipulation is challenging owing to the lack of depth information^66–68^. Depth sensing can be achieved using a force sensor to touch a target^69^. Piezoresistive AFM cantilevers have been applied for height estimation^70^. A vibrating piezoelectric bimorph actuator^71^, in which the vibration amplitude decreases upon contact with a substrate or target, was also demonstrated. Because force sensors are difficult to integrate with nanomanipulation tools, an image-processing-based approach was developed^72^. In this approach, after the nanomanipulator tool contacts the target substrate, lowering of the tool causes horizontal sliding, which is detected by image processing. After the relative vertical position of the nanomanipulation tool relative to the target substrate is detected, closed-loop position control along the *Z*-direction can be conducted for depth control.

Vision-based depth sensing was also realized by installing an optical microscope and camera to the SEM chamber wall for detecting tool tip depth^73,74^. This method’s detection resolution is limited by the Abbe diffraction limit of optical microscopy (that is, hundreds of nanometers). Stereo imaging was employed to determine tool depth by tilting either the SEM sample stage^75,76^ or the electron beam^67^. Mechanical components in stereo methods must be manufactured with tight tolerances, and image drift compensation must be carefully performed to achieve accurate depth sensing.

#### Force sensing

Force sensors provide force feedback in nanomanipulation^77,78^. Microelectromechanical systems (MEMSs) capacitive force sensors utilize comb structures to detect capacitance changes that are induced by externally applied forces^79,80^. A two-axis MEMS capacitive force sensor^81,82^ and an improved six-axis capacitive force sensor^83^ were constructed via deep reactive ion etching of silicon on insulator (SOI) substrates. These capacitive force sensors are capable of measuring forces from a few nanonewtons to micronewtons.

MEMS piezoresistive force sensors, which are formed by ion implantation, have also been developed for nanoscaled applications^77,79^. Piezoresistive force sensors were mounted on a nanomanipulator to provide high-accuracy force measurements that range from nanonewtons to millinewtons^84^. By using piezoresistive force sensors, mechanical indentation for stiffness determination of 2D materials and scanning for surface topography were achieved^45^.

When AFM probes are used for force sensing, an applied force can be determined from Hooke’s law^85,86^. The stiffness of an AFM probe can be calibrated using a reference cantilever of a known stiffness^87^. Image processing can be conducted to measure AFM probe deflections and determine the applied force. In^88^, vision-based force sensing was achieved using a deformable template-matching algorithm to visually determine the force distribution acts on a linearly elastic object using the contour information in an image. The effectiveness was validated using both AFM probes and microgrippers.

### Nanotools and tool exchangers

A number of nanotools are employed as end-effectors to perform nanomanipulation tasks, such as AFM probes, tungsten needles, and MEMS grippers. AFM probes and tungsten needles are the most popular end-effectors for nanomanipulation owing to their versatility and commercial availability. A nanomanipulator that is equipped with an AFM probe can perform topography imaging, indentation, stretching, cutting and pushing, pick-and-place operation and assembly, and nanolithography^2,8,40,70^. The AFM cantilever structure can be modified by FIB etching and deposition to create various types of application-specific tools. For instance, AFM probes were modified to have a flat tip^86,89^, stiff needle tip^87,90–93^, knife blade tip^94^ and fork tips^95,96^ for cellular and intracellular characterization and cell manipulation. Tungsten probes are fabricated and sharpened by electrochemical etching to obtain a high-aspect ratio. They were employed to perform tensile tests for mechanical characterization^97^ and pick-and-place assembly^98^, as well as electrical characterization of nanoscaled semiconductor devices and nanomaterials^26,30,99^.

Compared with these single-ended tools, MEMS grippers can be precisely actuated with large forces to effectively overcome material-substrate adhesion and perform reliable grasping of nano objects^100^. MEMS grippers are typically driven by electrostatic, electrothermal, and piezoelectric actuators^47,77^. They are made with feature structures using different fabrication methods^101–105^, and have been applied for various nanomanipulation tasks^106–112^, as summarized in Table 2. Although electrostatic actuation has low power, it requires relatively high actuation voltages. Electrothermal microgrippers operate under low voltages, produce short travel ranges, and can induce thermal drift to surrounding materials. The piezoelectrically driven microgrippers that are reported in Refs. 105,113–115 utilized piezoelectric bimorph structures and produced a motion range greater than 20 μm at high actuation voltages. Sensors can also be constructed in microgrippers for force-controlled grasping^116^. Tool exchangers have also been developed for easy replacement of broken tools and the modification of different types of end tools without opening the high-vacuum chamber of an SEM. A toolbox array with different tip morphologies, which are termed Nanobits, was fabricated by electron beam lithography and silicon processing^39,117^. A microgripper detached these tools from the tool array and assembled them to AFM probes or other end-effectors to form scanning probe tips^112,117^. The commercially available Oxford Instruments OmniProbe 400 system also features an *in situ* tool exchange and probe tip sharpening for repairing or replacing broken tools.

### Control

**Figure 2:**
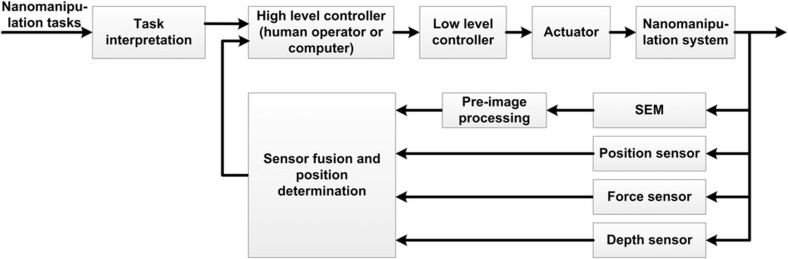
Control architecture of nanorobotic manipulation inside an SEM; scanning electron microscope.

For nanomanipulation inside an SEM, a look-and-manipulation scheme can be implemented to accomplish closed-loop control, as depicted in Figure 2. A high-level controller is responsible for supervising tasks, such as target tracking, trajectory planning, error handling, and the parallel execution of subtasks. With multiple types of sensing modalities (for example, force sensing, depth sensing, and positioning sensing), a sensor fusion approach can be employed to enhance the success rate of nanomanipulation and provide a basis for high-level control, decision-making, planning and fault-tolerance handling. A low-level controller receives commands from a high-level controller to generate driving signals for nanopositioning/nanomanipulation.

The low-level controller can be implemented via feedback, feedforward, or feedback–feedforward control^61,118,119^. A feedforward controller determines control signals according to the knowledge and modes of hysteresis, creep, and vibration. Because feedforward control does not rely on sensor feedback for high-bandwidth nanopositioning, the advantages are low cost and low hardware complexity^119,120^. In addition, techniques such as input shaping can be utilized to mitigate vibration^121^. However, the accuracy of feedforward techniques is dependent on both the model and the parameters identified in the model, which can change over time, especially in dynamic scenarios and the vacuum environment inside an SEM^122^. Feedback control utilizes sensor feedback, relaxes modeling requirements, and achieves better performance in terms of accuracy, vibration suppression, and uncertainty/disturbance rejection^61^. However, feedback-controlled nanopositioning has low bandwidth and requires sensor integration in the hardware platform. Therefore, feedforward- and feedback-controlled nanopositioning were attempted for higher bandwidth and higher accuracy^30^.

To achieve nanoscaled manipulation and assembly tasks, control approaches have been developed for tele-operated and automated nanomanipulation^49,123,124^. In tele-operated nanomanipulation, haptic and visual feedback are usually acquired and presented to a human operator. The operator sends task commands to the nanomanipulators via a joystick or a macromanipulator. Virtual reality techniques have been introduced to enhance this human-in-the-loop control system by enabling the operator to feel immersed in the environment based on various sensory cues^125,126^. Teleoperation involves significant human intervention and requires significant operator skills. Tele-operated nanomanipulation is slow and exhibits poor repeatability.

For automated nanomanipulation, real-time SEM visual feedback is important for providing visual guidance and realizing closed-loop control. To circumvent challenges such as SEM image noise and drift, image denoising and drift compensation methods were implemented using graphics processing unit (GPU) techniques^26,127^. SEM tracking algorithms can be classified into feature-based methods, model-based methods and hybrid methods^88,128–132^. They are commonly employed to provide a nanomanipulation system with visual feedback for automated operation.

### Electron-beam-induced deposition-assisted techniques

Electron-beam-induced deposition (EBID) is extensively applied inside SEMs as an important technique to deposit materials for welding and assembly at the nanoscale^42,133–135^. EBID involves the introduction of precursor gases into an SEM chamber from a nozzle after vaporization or sublimation. The gas molecules are subsequently irradiated by high-energy electrons, which decompose precursor molecules by secondary electrons that are diffracted from the irradiation spot and cause the deposition of nonvolatile fragments^136^. Several precursors are available for EBID to deposit various metals, dielectrics, and semiconductor materials, which render this technology a useful assistive technology for nanomanipulation, such as bonding end-effectors with materials^50,137^ for pick-and-place operation, soldering different materials for characterization^137^ and assembly^42,134^, and sensor instrumentation^138–140^. EBID can also be employed to remove materials when oxygen gas is introduced as a precursor^141,142^. EBID and nanomanipulation were employed to produce high-purity and hybrid metallic nanowires^139,143^, and various types of nanowires were grown with the introduction of different precursors. For instance, use of a nanomanipulator to precisely control the distance between a multi-walled CNT (MWCNT) field-emitter cathode and a tungsten probe emission anode, a high-purity platinum nanowire for intracellular PH sensing was formed using EBID with the introduction of trimethylcyclopentadienyl platinum (CpPtMe_3_) as a precursor^139^.

### Hybrid system integration inside SEMs

The large chambers of SEMs enable other microscopy and manipulation instruments to be integrated and form a hybrid system. These hybrid systems include AFM/SEM, AFM/FIB/SEM, STM/SEM, AFM/ESEM, and SEM/TEM, which take advantage of the strength of each tool for performing characterization and nanomanipulation tasks. Table 3 summarizes and compares these hybrid systems.

SEM and AFM are complementary techniques for performing topography and morphology measurements^16,144,145^. The integration of an AFM inside an SEM enables simultaneous imaging and manipulation in real-time to perform SEM-guided topography analysis with high-resolution and force feedback. FIB has also been integrated for material etching and deposition. Several hybrid AFM/SEM and AFM/FIB/SEM systems have been developed^28,40,43,146,147^, and commercial systems have also become available (for example, Semilab, Attocube, Trioptics, Nanonics Imaging Ltd, Kleindiek Nanotechnik).

**Figure 3:**
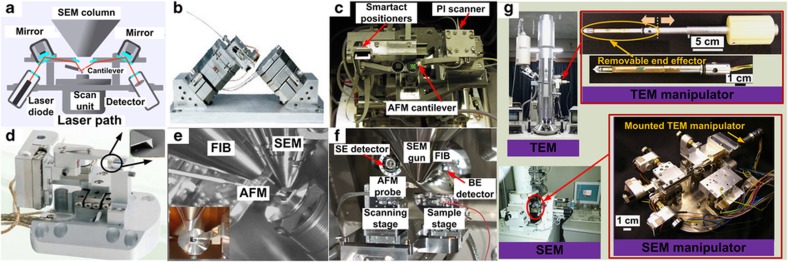
Hybrid system integration inside an SEM. (**a**) A hybrid AFM/SEM system based on laser beam deflection by DME-SPM. (**b**) AttoAFM/SEM system with a fiber-optic configuration by Attocube Systems AG. (**c**) A hybrid AFM/SEM system using self-sensing piezoresistive cantilevers. Adapted from Ref. 43. (**d**) An AFM system in dynamic mode for SEM integration by Trioptics. (**e**) 3TB4000 AFM/FIB/SEM system from Nanonics Imaging Ltd. (**f**) A hybrid AFM/FIB/SEM system. Adapted from Ref. 40. (**g**) A hybrid SEM and TEM manipulation system. Reprinted with permission from Ref. 13. AFM, atomic force microscope; FIB, focused ion beam; TEM, transmission electron microscope.

Conventional AFM is based on laser beam deflection and is not typically integrated inside SEMs owing to space and optical path constraints. A commercial hybrid AFM/SEM system by DME-SPM Semilab was designed with modified laser paths inside an SEM, as shown in Figure 3a. Attocube Systems AG uses a fiber-optic configuration to construct an *in situ* AFM for operation inside an SEM (attoAFM/SEM) with a laser interferometer (Figure 3b). For these laser-based methods, laser alignment must be carefully performed, and low laser power must be maintained to mitigate thermal drift in the vacuum chamber of an SEM.

Conversely, laser-free AFM that uses self-sensing cantilevers and tuning forks can be readily integrated inside an SEM. The AFM/SEM system reported in Ref. 43 employed piezoresistive cantilevers to perform scanning and manipulation tasks with force feedback, as shown in Figure 3c. Dynamic AFMs that use a tuning fork with a QPlus or Akiyama probe were also integrated inside an SEM to scan samples that are especially susceptible to surface damage^148^. A dynamic AFM was integrated inside an SEM by Trioptics to realize surface topography with a large scanning area of 500 by 500 μm (Figure 3d). The 3TB4000 system from Nanonics Imaging Ltd. is an instrument that integrates an AFM, which is based on a tailor made Q-Plus tuning fork, an SEM and an FIB, as shown in Figure 3e. This hybrid instrument enables imaging with a large field of view from an SEM, three-dimensional (3D) material and end tool modifications with FIB, and high-resolution AFM imaging. Figure 3f shows another hybrid AFM/FIB/SEM system that uses piezoresistive cantilevers in contact mode AFM imaging and manipulation with SEM imaging and FIB etching/deposition^40^.

STM/SEM integrated systems have also been developed for simultaneous imaging, manipulation, and measurements^149,150^. A hybrid system that consists of a four-probe STM and an SEM that is coupled to a molecular-beam epitaxy sample preparation chamber was developed for four-point electrical measurements and nanomanipulation of individual atoms to nanowires^150,151^.

Both TEMs and SEMs work in high-vacuum conditions. Therefore, water-containing samples cannot be directly observed inside an SEM or TEM^152,153^. Environmental scanning electron microscopes (ESEMs) overcome this limitation and permit the observation of liquid-phase materials, such as biological cells without metal coating and other electrically insulating materials that use a special secondary electron detector^86,154^.

The first hybrid AFM/ESEM system was developed for accurate topography measurements and tip–sample interaction observations^52,155^. Although ESEM’s imaging resolution is typically limited to a few nanometers, the integrated AFM achieved a resolution better than 0.2 nm in both contact mode AFM imaging and noncontact mode AFM imaging^52^. The nanomanipulation system that was reported in Ref. 90 was constructed inside an ESEM, which consists of two units for manipulation with seven DOFs and one cooling stage for holding samples and sample temperature control. This system performed simultaneous real-time observation and manipulation of biological samples for cell property characterization and surgery^87,91,95,156^.

To prepare the TEM samples, a hybrid nanomanipulation system that was integrated with an eight-DOF manipulator and a six-DOF manipulator inside an SEM and a TEM, respectively, was constructed^3,13,153^, as shown in Figure 3g. Samples were manipulated and prepared by the SEM manipulator onto the TEM manipulator/holder inside the SEM chamber and subsequently transferred to the TEM for observation and measurement^153^.

## State-of-the-art applications

This section discusses the applications enabled by nanomanipulation inside an SEM, including the characterization of the mechanical and electrical properties of nanoscaled materials and structures, the assembly of nanodevices (for example, biochemical sensors and nanoelectronics and nanophotonics devices), single-cell manipulation and subcellular organelle extraction, and 3D nanoscaled structural reconstruction of organelles.

### Manipulation and characterization of nanomaterials

**Figure 4:**
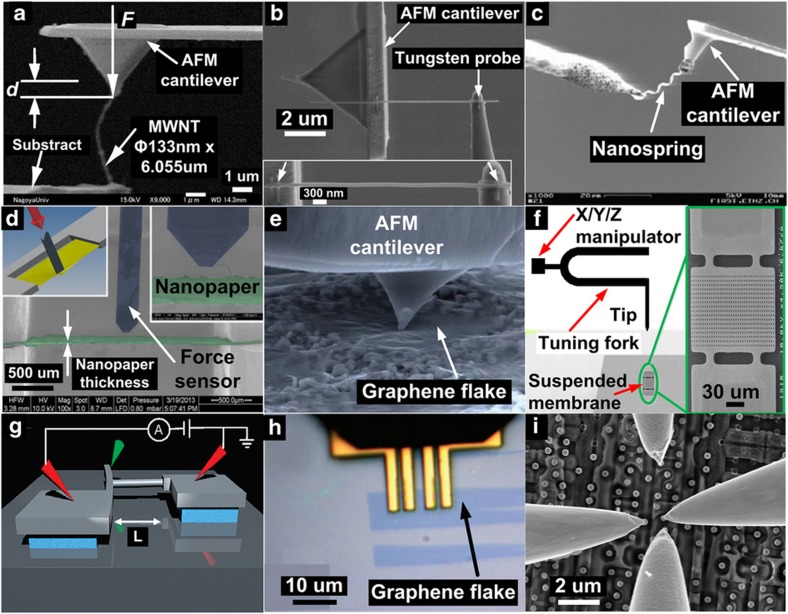
Examples of applications involving mechanical and electrical manipulation and characterization of nanostructures. (**a**–**c**) Mechanical characterization of MWNTs, nanowires and nanosprings. Adapted from Refs. 42,51,157. (**d**–**f**) Mechanical characterization of 2D materials of nanopapers, graphene films and suspended InP membranes using nanoindentation and contactless measurements. Adapted from Refs. 148,159,160. (**g**–**i**) Electrical characterization for nanowires, graphene flakes and single transistors on IC chips. Adapted from Refs. 28,162.

An individual MWNT was EBID-fixed on an AFM cantilever via nanomanipulation to determine the MWNT’s Young’s modulus^42^, as shown in Figure 4a. Mechanical characterization of one-dimensional nanomaterial was also conducted via tensile testing by two AFM cantilevers^50,137^. Using this approach, Zhu *et al.* conducted *in situ* tensile testing of a silver nanowire for mechanical characterization of its Young’s modulus, yield strength, and ultimate tensile strength^51^, as shown in Figure 4b. An InGaAs/GaAs nanospring was stretched for tensile tests using a similar method to determine its stiffness^157,158^ (refer to Figure 4c).

Figure 4d shows an example of the characterization of 2D nanomaterials, where 2D nanopaper composed of microfibrillated cellulose was fixed on both ends and driven against a capacitive force sensor probe to perform nanoindentation^159^. Figure 4e shows the indentation of a graphene film using a piezoresistive AFM cantilever. The graphene film was transferred to be suspended on a standard aluminum TEM grid^28,45^. Characteristic force-displacement curves were collected during the indentation process, and the Young’s modulus of the graphene films was determined^160^. Figure 4f shows non-destructive measurement using a tuning fork-based end-effector on a batch of suspended InP membranes to determine their stiffness values^148^. The tuning fork with a QPlus probe was controlled in frequency modulation mode, and the frequency shift was measured to calculate the sample stiffness^161^.

Nanomanipulation was also applied to conduct electrical characterization of nanomaterials. Figure 4g illustrates piezoresistivity characterization of a Si nanowire under tensile strain. The Si nanowire’s two ends were anchored on a cantilever and an insulated Si pad, respectively. One probe, which was labeled in green in Figure 4g, was used to apply stress to the freestanding cantilever, which generated strain on the Si nanowire. The two probes labeled in red were controlled to form electrical connections to measure the nanowire’s electrical resistance changes^162^. For the electrical characterization of graphene, an L-shaped four-point probe was fabricated using FIB and controlled to probe a graphene film^28^, as shown in Figure 4h. Nanoprobing inside SEM was also employed to attain I–V data of single transistors on IC chips by Kleindiek Nanotechnik (refer to Figure 4i), for identifying faulty locations and understanding failure mechanisms^27^.

### Assembly of nanodevices

**Figure 5:**
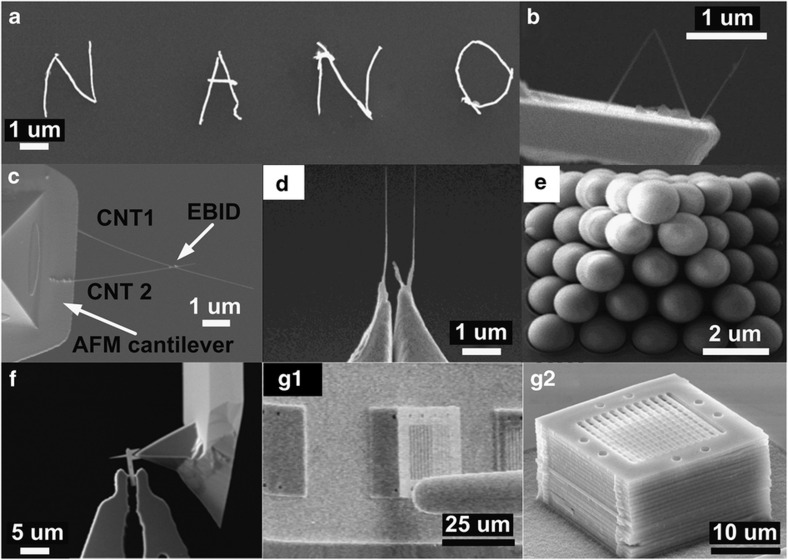
Examples of nano device and nanostructure assembly. (**a**) Nanowires were picked and placed to assemble a nano pattern. Adapted from Ref. 163. (**b**) A MWCNT was formed using a 3D letter with EBID. Adapted from Ref. 13. (**c** and **d**) A thermal sensor and a pair of nanoscaled tweezers were assembled with nanomanipulation. Reprinted with Institute of Physics Publishing (IOP) permission from Ref. 138 and adapted from Ref. 165. (**e**) 3D pyramidal spheres were assembled. Adapted from Ref. 70. (**f**) A nanotool was mounted on an AFM cantilever. Reprinted with IOP permission from Ref. 39. (**g**1 and **g**2) Assembly of photonic plates to form a 3D photonic crystal. Adapted from Ref. 1. EBID, electron-beam-induced deposition; MWCNT, multi-walled CNT.

With its precise positioning and manipulation capabilities, nanorobotic manipulation has enabled the assembly of nanostructures and nanodevices. Figure 5a shows that individual gold nanowires were picked and placed by two nanoprobes and subsequently welded and assembled to form the nano pattern ‘NANO’^163^. A 3D letter ‘N’, as shown in Figure 5b, was formed by EBID with oxygen as a precursor for cutting and bending of a MWCNT and nanomanipulation for assembly^13,141,164^. Figure 5c shows a thermal sensor that is composed of two MWCNTs assembled on an AFM cantilever with EBID and nanomanipulation^138^. In Figure 5d, two nanotubes were successively manipulated and assembled to form a pair of nanoscaled tweezers^165^. A DC voltage was applied to open and close the nanotweezers for the pick-and-place of nanoparticles.

A 3D pyramidal structure that consists of stacked silica spheres, as shown in Figure 5e, was assembled via cooperative manipulation using two nanoprobes. A tungsten nanoprobe and a modified piezoresistive AFM cantilever with a spherical adhesion pocket tip were employed to pick and place the silica spheres^70^. In Figure 5f, a custom nanotip was manipulated by a microgripper to approach an AFM cantilever and subsequently soldered and assembled onto the AFM cantilever tip using EBID to construct a high-aspect-ratio AFM cantilever tip for high-resolution imaging^39^. To produce a 3D photonic crystal device (Figure 5g2)^1^, a thin plate was separated from a substrate after the connection bridge was broken with a nanoprobe and picked and transferred for assembly, as shown in Figure 5g1.

Nanorobotic manipulation also enabled the assembly of a variety of other NEMS devices^7,22,166,167^ for applications in the nanoelectronics and bionanotechnology sectors, as summarized in Table 4.

### Cell characterization and manipulation

**Figure 6:**
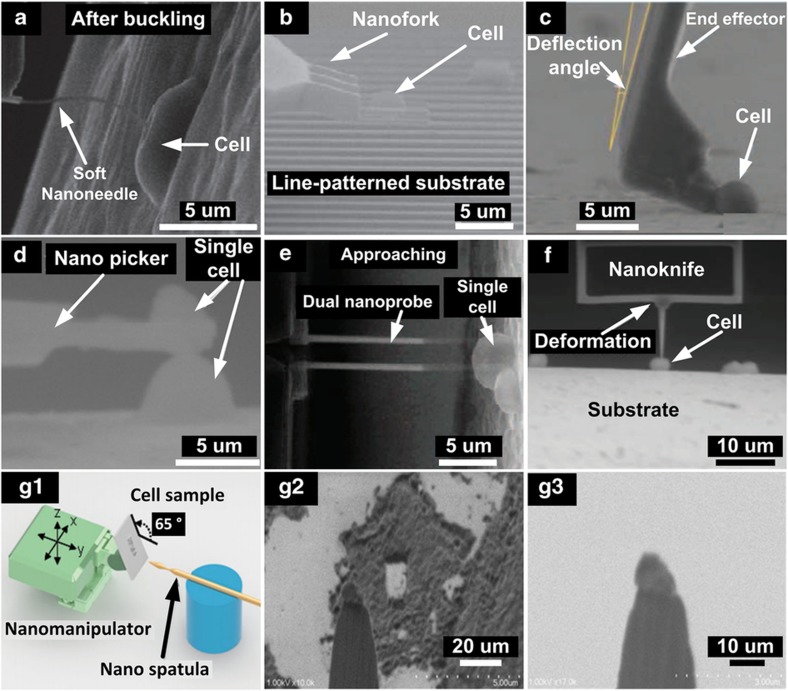
Cell characterization and manipulation inside an ESEM and SEM. (**a**) Cell stiffness measurement. Adapted from Ref. 90. (**b**–**d**) Cell–substrate and cell–cell adhesion force determination. Adapted from Refs 89,95 and reprinted with IOP permission from Ref. 96. (**e**) Intracellular electrical measurement for viability testing. Adapted from Ref. 36. (**f**) Single cell cutting. Reprinted with IOP permission from Ref. 94. (**g**1–**g**3) Chromatin extraction process. Adapted from Ref. 37.

Standard AFM cantilevers were modified using FIB etching and deposition to produce different types of functional tools, such as a soft buckling nanoneedle^86,90,91^, a nano-fork and a nano putter^95,168^, as shown in Figures 6a, b and c, respectively. These tools were mounted on a nanomanipulation system in ESEM to perform indentation to determine cell stiffness and viscoelastic properties^87,90^. The nanomanipulation system was also employed to lift and push a cell to measure adhesion forces between the cell and substrate^89,95,156,168^; to measure cell–cell adhesion force^96^; and to electrically characterize intracellular properties^36^, as shown in Figures 6d and e. Figure 6f shows the use of a nano-knife for performing cell surgery of a yeast cell^94^. The extraction of DNA from a single-cell nucleus was performed via nanomanipulation under SEM. A nanomanipulation system equipped with a nanospatula as the end-effector was employed to dissect and collect a single-chromatin complex from within a cell nucleus after correlating SEM images and fluorescence microscopy images to determine the target locations to extract^169^, as shown in Figures 6g1–g3. Because gene locations within the nucleus are not random, this technique enables high-throughput gene mapping for exploring gene loci associations with nuclear substructures^37^.

### Automated nanomanipulation

Manual nanomanipulation by a joystick is time-consuming and skill dependent. Over the past decade, progress in automated nanomanipulation tasks has been achieved^49,170^. Table 5 summarizes representative automated nanomanipulation. The majority of automated SEM-based nanomanipulation tasks were performed with custom instruments using piezoelectric positioners. AFM cantilevers, tungsten probes and MEMS grippers with EBID-assisted soldering techniques are employed for assembly and pick-and-place operation, primarily via SEM-based visual servoing and assisted by force and depth detection. Developing the non-application-specific automated nanomanipulation solutions remains challenging.

#### Automated pick-and-place of nano objects

In Ref. 112, a MEMS gripper was controlled to automatically pick up a CNT from a substrate and place and solder it onto a target structure. Depth-from-focus and depth detection using a piezoelectric touch sensor were employed for coarse and fine alignment to place the microgripper with respect to the CNT in the *Z*-direction for pick-up^71^. A manipulation strategy using line and two-point contact to adjust adhesion forces was developed to place the CNT onto a target AFM tip to form a CNT-enhanced, high-aspect-ratio AFM tip for scanning deep trenches^112^. For mechanical characterization, individual silicon nanowires were automatically picked up and placed on a MEMS tensile testing device^98,171^. Via visual recognition and vision-based closed-loop control, a nanowire was picked up from the growth substrate and moved for fixation on the MEMS device with EBID. Automated pick-and-place of individual colloidal spheres was realized using two nanoprobes with tailored geometries for cooperative manipulation to form a 3D pyramidal structure via visual servoing and force control^70^. The method did not involve the use of EBID and provided a flexible strategy for 3D nano assembly.

#### Automated nanoprobing

**Figure 7:**
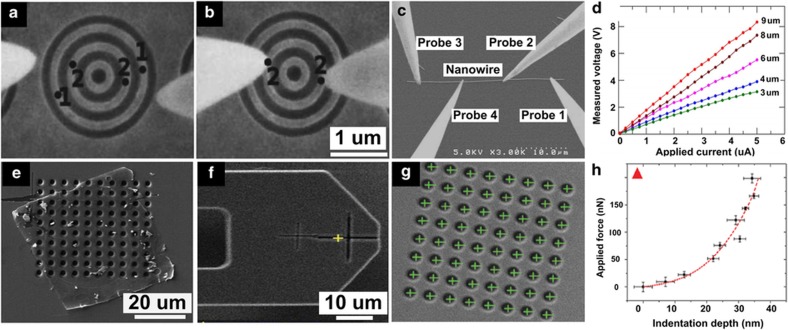
Automated nanomanipulation. (**a** and **b**) Automated nanoprobing. Adapted from Ref. 26. (**c** and **d**) Automated four-point probe measurement on a single nanowire. Adapted from Ref. 30. (**e**–**h**) SEM-guided AFM stiffness measurement of graphene. Adapted from Ref. 45.

Nanomanipulation is capable of automated positioning of nanoprobes on nanostructures to perform electrical characterization for fault analysis and quality control. Automated nanoprobing was performed to probe nanostructures on SEM metrology chips, as shown in Figures 7a and b. Algorithms were developed on a GPU to realize real-time SEM image denoising and drift compensation, which enable robust visual tracking and visual servo control for automated nanoprobing^26,27^. Automated four-point probe measurements on single nanowires were also performed for electrical characterization^30^, as shown in Figure 7c, where four tungsten nanoprobes were moved downward to contact the substrate via a vision-based contact-detection method^72^ and subsequently moved upward to a certain height above the substrate. Via visual servo control with a feedforward controller^69,172^, the four probes were positioned via closed-loop control to land on their respective target locations along the nanowire at a pre-defined separation distance. The I–V characteristics of the nanowire with regard to different separation distances between the two inner probes are shown in Figure 7d.

#### SEM-guided, automated AFM manipulation

SEM imaging can be employed to guide the AFM cantilever to localize the regions of interest where AFM imaging or measurement is conducted. A hybrid AFM/FIB/SEM system that employed a piezoresistive cantilever was controlled to realize automated indentation for mechanical characterization of graphene membranes^45^. The piezoresistive cantilever provided force feedback, and a calibration cross-structure by FIB milling was created on its back surface to enable reliable visual tracking of the cantilever. Graphene membranes were automatically picked up and transferred to suspend on a substrate with a grid pattern, as shown in Figure 7e. SEM imaging was utilized to detect the cantilever position and the centers of holes in the pattern and guide the cantilever to perform nanoindentation at the detected centers (Figures 7f and g). Indentation depths and applied forces, as shown in Figure 7h, were measured for determining the Young’s modulus values of the graphene membranes.

### 3D image reconstruction by nanotomography

Ultramicrotomy involves serially cutting and imaging thin slices of a sample under an SEM. It has served an important role in studying the anatomy of cells and tissues in histology and tomography in materials science^173^. In 2004, Denk and Horstmann presented serial block face scanning electron microscopy (SBFSEM)^174^, which is a nanomanipulation method for automatically sectioning and 3D reconstructing of tissue structures. The microtome inside an SEM consisted of a moving diamond knife for slicing a sample and an actuator/positioner to advance the specimen after each slice. After each slicing, the remaining block face was imaged^174^. Algorithms for image segmentation and structural reconstruction were developed to generate a 3D tissue nanostructure of biological and other material specimens from the serial images with nanometer resolutions^76^.

The development of SBFSEM has enabled advances in neuroscience, in which previous studies have either focused on detailed small volumes or averages over large volumes. SBFSEM bridges the gap by enabling the tracing of neuronal networks and revealing synaptic connections with nanometer resolutions over volumes as large as 1 mm^3^ (Ref. 175). The technique is sufficiently fine to resolve morphologies of structures, which can provide clues to their function for a better understanding of certain pathologies. Study tissues that involve long tissue fibers can particularly benefit from SBFSEM. The technique has also been applied in materials science to study the microstructure of engineered materials^176^.

## Discoveries enabled by SEM-based nanomanipulation

Leveraging the technical advances in SEM-based nanomanipulation, many fundamental discoveries have been made, some of which are summarized in Table 6. Mechanical characterization of nanomaterials has significantly benefited from SEM *in situ* nanomanipulation. For instance, nanomanipulation and tensile testing of Ag nanowires of different diameters revealed quantitatively the size and structure effects on the nanowire’s mechanical properties (that is, Young’s modulus, yield strength, and ultimate tensile strength)^51^. Nanomanipulation was also conducted to perform measurements on silicon nanowires^177^ and CNTs^50,137^ to understand how their properties change with size and structure variations. Nanomanipulation and mechanical measurements also shed light on the degradation process of silicon nanowire anodes in lithium-ion batteries^178^, contributing to the optimization of battery electrode design for enhanced reliability.

Nanomanipulation and electrical characterization of strained Si nanowires revealed the positive piezoresistive effect at low strain levels and also found carrier mobility enhancement for strained Si-CMOS (silicon-complementary metal-oxide semiconductor) in semiconductor devices^162^. At high-strain levels, the negative piezoresistive effect became apparent, and no fatigue failures occurred after several hundred loading cycles. These findings emphasize the importance of crystallinity and strain for Si nanowires in semiconductor applications^162^.

SEM *in situ* electrical characterization of GdSi_2_ quantum nanowires explained how the electronic transport nature is modified by local atomic defects and interwire coupling in a quantum wire system^179^. The atomic defects produce electron localizations in isolated nanowires, and interwire coupling stabilizes the structure and promotes the metallic states in wire bundles^180^. For IC testing at the transistor level, electrical characterization by nanoprobing inside an SEM was performed to evaluate SRAM cells for failure analyses, which revealed the root causes and failure mechanisms^27^ and contributed to the packaging and IC-MEMS integration^181^.

Intracellular dissection via nanomanipulation inside an SEM extracted target DNA from a single-cell nucleus to discover gene associations with nuclear bodies^37^. Four new gene loci on chromosomes 11, 17, and 18 that have a significant association with promyelocytic leukemia nuclear bodies, which are tumor-suppression proteins in humans, were discovered. Known gene loci on chromosomes 1 and 6, which are associated with histone locus bodies, were reconfirmed; this finding proves that nanodissection is a viable method for discovering mechanisms for nuclear event regulation. Nanomanipulation of cellular materials revealed how chemical bonds regulate cell–cell adhesion and its contact-time dependence^96^.

SBFSEM was employed to study neuronal circuit development^182^. When new synapses are formed, they are innervated by many axons. During development, the connections carry competing signals, and the axon with the strongest signal remains connected, while the remaining axons withdraw in a process that is referred to as synaptic pruning or synapse elimination. Using SBFSEM, the postsynaptic membrane of an axon that carries the ‘winning’ signal was reinforced. SBFSEM was also employed to investigate the role of bone morphogenetic proteins (BMPs) in synapse generation and development^183^. In the absence of BMPs, synapse elimination at the calyx of Held in a mouse brain did not occur normally, whereas control mouse neurons undergo normal synapse elimination in the presence of BMPs. Studies have suggested that abnormal synapse elimination may be a risk factor for neurological or psychological diseases^184,185^. The structure of podocytes, which is a type of cell in kidneys that filters blood^186^, were also examined using SBFSEM. Three different structures, including the cell body, the primary process, and the foot process, were previously identified. However, the SBFSEM data discovered ridge-like structures on which the foot processes are anchored. These ridge-like structures appear on both the primary process and the cell body; this discovery changes a misunderstanding of podocyte anatomy.

## Summary and outlook

This paper presented technical advances in the establishment of nanorobotic manipulation laboratories inside SEMs for simultaneous imaging and nanomanipulation. These platforms and nanomanipulation techniques have enabled the *in situ* characterization of nanomaterials, the assembly of nano device prototypes, and the analysis of subcellular organelles. Despite the significant progress that has been achieved in the past two decades, challenges remain in the realization of 3D nanomanipulation with high precision, robustness, flexibility, and high throughput.

The majority of SEM-based nanomanipulation tasks are manually performed. Although a number of sensing technologies have been developed for integration into nanomanipulation systems, automation remains dependent on SEM imaging as feedback. The low frame rate of SEMs and the high noise, drift, and distortion of real-time SEM imaging hinder the achievement of reliable visual tracking and pose estimation of end-effectors and target objects for high-speed nanomanipulation. Advanced visual tracking methods must be developed to cope with blurred and distorted SEM images. Advanced control schemes that employ integrated information from image-, force-, depth-, and position-sensing modalities are needed for effective decision-making, planning, and manipulation.

During the past decade, efforts have been made in automated nanomanipulation (for example, pick-and-place of CNTs^44^ and nanowires for mechanical characterization^98^, nanowire field-effect transistor assembly^32^), nanoprobing for electrical characterization of nanowires and transistors^26,30^, and SEM-guided AFM manipulation for the transfer and stiffness measurement of graphene membranes^28,45^. Automated nanoprobing has demonstrated higher consistency and at least three times faster operation than manual operation^26^, in addition to minimizing the risks of nanotools and sample breakage^30^. Automated pick-and-place of nanowires was completed within 10 min compared with 2 h by teleoperation^98,171^. Although techniques such as visual tracking, depth detection, and the integration of feedback and feedforward control for piezoelectric positioning have been developed for automated nanomanipulation in known environments, system performance may deteriorate in changing environments with uncertain physical parameters and dynamic disturbances.

The application of nanomanipulation has penetrated several disciplines and sectors, such as materials science, semiconductor, cell biology, and neuroscience. Some notable achievements are mechanical and electrical characterization of graphene membranes^28,45^, electrical measurement of single transistors^27^, 3D transfer of graphene^28,45^, assembly of photonic crystal devices^1,187^, identification of the size effect on the mechanical properties of nanowires and the strain effect on the piezoresistive properties of nanowires^51,162^, discovery of new gene loci associated with promyelocytic nuclear bodies^37^, and tracing of neuronal networks and synaptic connections^182,183^.

The formation of hybrid systems by integrating other instruments into an SEM has also produced unique capabilities. For instance, STM/SEM integration is capable of manipulating individual atoms; characterizing electrical transport of CNTs, bending, and cutting nanofibers; and fabricating nanowires^151,188^. This integration has also enabled fundamental discoveries of intrinsic structure-transport at the atomic scale^180^ and the effect of applied stress on dynamic phase evolution^189^. In addition to STM, nanoscale laboratories inside an SEM have also integrated AFM, FIB, optical microscopes, and multi-tool changers. Further advances in hardware development will produce powerful *in situ* capabilities for manipulation, assembly, and characterization of nanoscales objects and materials to close the gap between current bottom–up and top–down technologies.

With the unique advantages of programmability, automation, and specificity, nanomanipulation inside an SEM will continue to serve as a strong driver of scientific discoveries and further evolve into a more powerful workhorse technology for the nano sciences and nanotechnology industries.

## Figures and Tables

**Table 1:** Summary of commercial nanomanipulation systems

System	Speed	Coarse motion range	Fine motion range	DOF	Coarse resolution	Fine resolution
Zyvex S100	3 mm s^−1^	12 mm XYZ	50 μm for XY; 10 μm for Z;	Four units with 24 axes	100-nm open loop	5-nm open loop
Xidex NanoBot NX-2000	1 mm s^−1^	12 mm XYZ	5 μm XYZ	Two units with six axes	50-nm open loop	1-nm open loop
Oxford Instruments OmniProbe 400	N/A	4 mm XYZ	Not specified	One unit (four axes with one rotation)	10-nm closed loop	N/A
TNI LifeForce	>10 mm s^−1^	10 mm XY 5 mm Z	30 μm XYZ	Four units with 12 axes	<100-nm open loop	0.1-nm open loop 1-nm closed loop
Kleindiek MM3A	2 mm s^−1^ telescoping; 10 mm s^−1^ rotation	12-mm telescoping; 240° rotation	Not specified	One translation Two rotations	5-nm open loop rotation; 0.5-nm open loop translation	N/A
SmarAct SLC	>10 mm s^−1^	10 mm XYZ	1.5 μm XYZ	Four units with 12 axes	20-nm closed loop	1-nm closed loop
Imina miBot BT-11	2.5 mm s^−1^	Unlimited XY 10 mm Z	440 nm XY 780 nm Z	Two translations Two rotations	40-nm open loop	0.5-nm open loop

Abbreviation: DOF, degrees of freedom.

**Table 2:** MEMS grippers that are used in nanomanipulation

Principle	Actuation structure	Fabrication	Actuation voltage	Motion range	Usage in nanomanipulation
Electrostatic driven	Comb drive	DRIE of SOI^101,102^	Medium voltage	~10 μm	Pick-and-place of nano spheres and micro particles^106–108^
Electrothermal driven	U-shaped and V-shaped beams	RIE of SOI^103^	Low voltage	~2 μm	Pick-and-place of nanowires^109,110^; assembly to form customized scanning probe tips^111,112^
Piezoelectric driven	Bimorph	Ultrasonic machining of piezoceramic plates^104,105^	High voltage	>20 μm	Manipulation of CNTs, micro grains and copper spheres^105,113–115^

Abbreviation: DRIE, deep reactive ion etching; SOI, silicon on insulator.

**Table 3:** Summary of hybrid systems

Hybrid systems	Configuration		End effectors	Functions	Limitations
AFM/SEM	Optics-based		AFM cantilevers	All available AFM modes (for example, DME-SPM Semilab, Attocube)	Thermal drift
	Optics-free	Contact mode	Piezoresistive cantilevers	Scanning and manipulation with force feedback^43^	Piezoresistive cantilevers only
		Dynamic mode	Tuning fork-based probes; Akiyama probe	Scanning for ultrasensitive surface topography^148^	No manipulation functions
AFM/FIB/SEM	Optics-free	Contact mode	Piezoresistive cantilevers	Fabrication, scanning and manipulation with force feedback^40^	Piezoresistive cantilevers only
		Dynamic mode	Tuning fork-based probes; Akiyama probe	Fabrication and surface topography (for example, Nanonics Imaging Ltd)	No manipulation functions
STM/SEM	Optics-free		Tungsten probes; Conductive AFM probes	Scanning with current feedback at both low and room temperatures^150,151^	Conductive probes only
AFM/ESEM	Optics-based		AFM cantilevers	All AFM modes available, especially for biological samples^52^	Thermal drift
SEM/TEM	TEM holder with piezo actuators		No limitations	Sample preparation in SEM, and manipulation in TEM^153^	Small workspace inside TEM

Abbreviations: AFM, atomic force microscope; SEM, scanning electron microscope; STM, scanning tunneling microscope; TEM, transmission electron microscope.

**Table 4:** Nanoelectromechanical systems (NEMS) constructed by nanomanipulation inside SEM

NEMS	Types		References
Nanotube-based	Nanoactuator	Linear motor	22,31,42
		Linear bearing	42,166
	Nanosensor	Thermal sensor	138
		Mass flow sensor	42
	Nanotool	Nanotweezers	165
		Nanoscissors	42
		SPM probes	112,133
		Field emitters	133,139
			
Nanowire-based	Nanosensor	Gas sensor	40
	Nanotool	Transistors	32,40,167
			
Graphene-based	Nanosensor	Oscillator	28

Abbreviations: NEMS, nanoelectromechanical systems.

**Table 5:** Summary of automated nanomanipulation

Task	Nanotool	Feedback	Object size	Success rate	Throughput	References
Pick-and-place of particles	Two end-effectors formed on an AFM cantilever	SEM-based visual servoing; capacitive position sensors; piezoresistive AFM cantilever for contact force detection	1160 nm, 519 nm, 237 nm	100% for 100 particles	112 s per particle	70
Pick-and-place of nanowire	Electrothermal MEMS gripper;	SEM-based visual servoing; piezo bimorph touch sensor for depth sensing	30–150 nm	Not reported	Not reported	71
	Tungsten probes	SEM-based visual servoing	74–113 nm	Not reported	10 min per nanowire	98
Pick-and-place for nanotool assembly	Electrothermal MEMS gripper; FIB modified AFM cantilever	SEM-based visual servoing; optical encoder	3–4-μm long, 150-nm thick	Not reported	Not reported	39
Nanoprobing of nanostructures	Tungsten probes	SEM-based visual servoing	130 nm	Not reported	15.3 s per 2 locations	26
Nanoprobing of nanowires	Tungsten probes	SEM-based visual servoing; vision-based contact-detection	70–100 nm	100% for 50 measurements	20 s per nanowire	30
Membrane indentation	Piezoresistive AFM cantilever	SEM-based visual servoing; piezoresistive indentation force sensing; capacitive position sensors	50-nm thick	Not reported	Not reported	45
Intracellular DNA extraction	Nanospatula	SEM-based visual servoing; strain gauges for position-sensing	100 nm	16%	Not reported	37

Abbreviations: AFM, atomic force microscope; FIB, focused ion beam; SEM, scanning electron microscope.

**Table 6:** Examples of discoveries enabled by nanomanipulation inside an SEM

Technique	Field of discovery	Discovery	References
Mechanical testing	Nanomaterials	Young’s modulus, yield strength, and ultimate tensile strength of Ag NWs increased as the diameter decreased. Yield strain scaled with surface area, and yielding was caused by dislocation nucleation from surface sources. The pronounced strain hardening was primarily attributed to the presence of internal twin boundaries.	51
	Lithium-ion battery	The delithiated Si nanowire exhibited a significant decrease in the elastic modulus and the ultimate tensile strength owing to the newly formed amorphous Si layers.	178
Electrical nanoprobing	CMOS manufacturing	Strained Si nanowires revealed the positive piezoresistance effect at a low strain level of <0.8%, whereas an anomalous negative piezoresistance effect and fatigue failure were not observed after several hundred loading cycles for high-strain levels.	162
	Nanomaterials	Large discrete resistance jumps were measured at the random grain boundaries (GBs) in copper nanowires. A metal−insulator transition is revealed in GdSi_2_ quantum nanowires, whereas a robust metallic state is obtained in wire bundles at low temperatures. The strain effect has served an important role dynamic phase evolution for both phase separation and Mott metal–insulator transition owing to strong electron-lattice coupling.	150,179,180
	Semiconductor	A variation in threshold voltage for each type of cell transistor was normal distribution; marginal failures or degradations that relate to the ultrathin gate oxides, variations in the resistance of the implanted layers in the substrate, and an abnormal passive-voltage-contrast signature were determined.	27
Cellular dissection	Cell biology	Four new gene loci were associated with promyelocytic nuclear bodies, which are tumor-suppression proteins in humans.	37
Cellular characterization	Cell biology	A time effect on yeast cell–cell adhesion force was observed: The force rose to approximately 25 nN with an increase in contact time for the first few minutes and subsequently attained a balance condition with constant force. The stiffness of a single cell decreases with increasing humidity.	93,96
SBFSEM	Neuroscience	Postsynaptic membrane of the predominant synaptic connections were reinforced with use to form a permanent connection, whereas other axons are pruned.	182
	Neuroscience	Synaptic pruning does not proceed normally in the absence of bone morphogenetic proteins, and synapses remain multiply innervated.	183
	Cell biology	A previously unknown ridge-like structure on podocytes was discovered, which changed the understanding of podocyte anatomy.	186

Abbreviations: CMOS, complementary metal-oxide semiconductor; NW, nanowire; SBFSEM, serial block face scanning electron microscopy.
